# Significance of Gastrointestinal Histiocytosis: A Case Series and Literature Review

**DOI:** 10.7759/cureus.110313

**Published:** 2026-06-05

**Authors:** Paige Gurizzian, Ridhima Kaul, Arjun Chatterjee, Renan Prado, Erna Forgo, John McMichael, Sudipto Mukherjee, Pauline Funchain, Brian Baggott

**Affiliations:** 1 Department of Internal Medicine, Cleveland Clinic Foundation, Cleveland, USA; 2 Department of Gastroenterology and Hepatology, Cleveland Clinic Foundation, Cleveland, USA; 3 Department of Pathology, Cleveland Clinic Foundation, Cleveland, USA; 4 Department of Hematology and Medical Oncology, Cleveland Clinic Foundation, Cleveland, USA; 5 Department of Medicine, Stanford University, Stanford, USA

**Keywords:** endoscopic biopsy, gastrointestinal histiocytosis, immunohistochemistry staining, langerhans cell histiocytosis (lch), langerhans histiocytosis

## Abstract

Background and aims

Gastrointestinal (GI) histiocytosis is an uncommon and heterogeneous finding that may represent reactive, infectious, medication-related, or neoplastic processes. Because lesions are often identified incidentally on biopsy, their true frequency, etiologic spectrum, and optimal diagnostic approach remain poorly defined. We aimed to characterize GI histiocytosis using a large institutional cohort and contextualize findings using a review of published adult cases.

Methods

We performed a retrospective case series of adult patients at the Cleveland Clinic Foundation with histiocytic involvement of the GI tract identified on pathology specimens between 2007 and 2022, along with a systematic review of adult cases published on PubMed from 2002 to 2022. Extracted data included demographics, presenting symptoms, indications for endoscopy, anatomic distribution, endoscopic findings, associated conditions, and available immunohistochemical and molecular results.

Results

The institutional cohort included 108 patients (mean age 63.7 years; 60.2% female), of whom 35% were asymptomatic. Involvement spanned the esophagus to rectum, most commonly affecting the stomach. Endoscopic findings ranged from normal mucosa to polyps, nodules, and ulcerations. Nearly half of cases lacked definitive etiologic classification.

Conclusions

GI histiocytosis may be more common than previously appreciated but frequently remains incompletely evaluated in routine clinical practice. Integrating clinical context with targeted histologic assessment and selective molecular testing may help distinguish incidental reactive findings from clinically significant histiocytic disorders.

## Introduction

Histiocytes are derived from macrophages and Langerhans cells and play an important role in the immune response to pathogens and promote tissue repair. In the gastrointestinal (GI) tract, histiocytes are typically located within the lamina propria, particularly in Peyer patches in the small intestine [1-3]. Histiocytic disorders of the GI tract involve the excessive infiltration of histiocytes, leading to a variety of symptoms including abdominal pain, diarrhea, weight loss, GI bleeding, and obstruction, depending on the severity and location of the histiocytes [1]. Histiocytic disorders of the GI tract can be divided into primary histiocytic disorders of uncertain origin, primary histiocytic disorders of neoplastic origin, reactive and infectious conditions, systemic diseases with secondary GI tract involvement, and pigmented histiocytic aggregates [1,3,4].

Primary histiocytic disorders of uncertain origin include Rosai-Dorfman disease, Langerhans cell histiocytosis (LCH), and Erdheim-Chester disease. Rosai-Dorfman is a benign condition that affects the cervical lymph nodes of pediatric patients [4]. Less than 20 cases of Rosai-Dorfman involving the GI tract have been reported. LCH can affect any organ in the body, although bone (80%) and skin (33%) are most commonly affected [3,5]. GI tract involvement is usually in younger patients with a more severe disease course [1,4,5]. BRAF mutations, particularly the V600E mutation, have been reported in 40-70% of LCH cases, and MAP2K1 mutations have been reported in BRAF-negative cases [6]. In addition to the mutations in BRAF and MAP2K1 genes, there is a distinct histiocytic subtype known to be associated with KIF5B-ALK fusions. This subtype is characterized by frequent neurologic involvement [7]. Erdheim-Chester disease is a multisystemic disorder that most commonly affects the bones, lungs, vessels, retroperitoneum, and CNS [8,9]. While Erdheim-Chester disease can involve various organs, GI tract involvement is extremely rare [8]. Primary histiocytic disorders of neoplastic origin include histiocytic sarcoma and follicular dendritic cell sarcoma. Histiocytic sarcomas occur in the GI tract, skin, and soft tissue and are usually aggressive tumors [4]. Follicular dendritic cell sarcoma is more indolent and usually present with lymphadenopathy, although it can involve the GI tract [4].

Reactive and infectious conditions that cause histiocytes in the GI tract include xanthomas, xanthomatosis, xanthogranulomatous inflammation, juvenile xanthogranuloma, muciphages, Whipple disease, intestinal *Mycobacterium avium* infection, and malakoplakia. The difference between xanthomas and xanthomatosis is based on size. Xanthomas (also known as xanthelasma) are collections of lipid-laden, “foamy” histiocytes in the mucosa [4]. Xanthomatosis is a larger xanthoma or mass-forming lesion [4]. Xanthogranulomatous inflammation is believed to be the result of changes over time of xanthomas resulting in fibrosis and sometimes giant cells [4]. Juvenile xanthogranuloma, as the name implies, classically presents as a yellowish nodule in infants and children. These typically occur in the skin; GI tract involvement is exceptionally rare apart from the liver [4,10]. Muciphages appear similar to xanthomas, but the histiocytes are rich in mucin; therefore, stain positive on Periodic acid-Schiff (PAS) special stain, whereas xanthomas are PAS-negative [4]. Whipple disease is an infectious disease caused by* Tropheryma whipplei *with the classic symptoms of diarrhea, arthralgia, and neurologic involvement [4]. Similar to muciphages, the intracytoplasmic granules in Whipple disease stain positive on PAS special stain. *M. avium* infection of the intestine is another PAS-positive histiocytic disease, although this can be distinguished from muciphages and Whipple disease as it will stain positive on Ziehl-Neelsen special stain [4]. Malakoplakia is a rare granulomatous disease which is believed to be caused by a defect in phagosome-lysosome fusion and the killing capacity of macrophages after endocytosis [11].

Systemic diseases with secondary GI involvement include Tangier disease and lysosomal storage diseases. Tangier disease is an autosomal recessive disorder characterized by high levels of lipoprotein and cholesterol, early atherosclerosis, hepatosplenomegaly, peripheral neuropathy, and yellow nodules or orange-brown spots on the intestinal mucosa [12]. Lysosomal storage diseases include Niemann-Pick disease, GM1 gangliosidosis, Wolman’s disease, and Fabry disease. In the GI tract, neuronal cells and phagocytic cells are usually affected [4]. Lysosomal storage diseases also stain positive on PAS special stain, in addition to Oil Red O and Sudan black special stains [13].

Lastly, pigmented histiocytic aggregates can be found in the GI tract. Melanosis coli, as the name implies, is a collection of macrophages with brown pigment in the colon caused by chronic laxative use [14,15]. Pseudomelanosis coli is a collection of macrophages, although these contain black pigment from iron and hemosiderin, and can be found in the duodenum and stomach [4]. Lastly, barium granulomas can occur in patients who underwent barium contrast radiography with extravasation of barium [16].

Despite this broad etiologic spectrum, GI histiocytosis is often encountered as an incidental biopsy finding, and the distinction between reactive histiocytic aggregates and clinically significant histiocytic disorders may not be clear in routine practice. As a result, the frequency, clinical associations, downstream evaluation, and etiologic classification of these findings remain poorly defined. Our study aimed to characterize the clinical, endoscopic, histopathologic, and etiologic features of GI histiocytosis using a retrospective case series of adult patients at the Cleveland Clinic Foundation (CCF) and a systematic review of the published literature.

## Materials and methods

This retrospective cohort study at our institution received approval from the institutional review board (IRB, #26-080) and utilized electronic medical records. We searched the CCF pathology database to identify adult patients with histiocytic involvement of the GI tract between 2007 and 2022, including sites from the oropharynx to the anus as well as the liver, gallbladder, and pancreas (Table 1). Extracted variables included patient demographics (age, sex, race), presenting symptoms, indication for endoscopy, anatomic site(s) of GI involvement, and endoscopic findings. Pathology reports were identified through the institutional pathology database, and data were extracted from finalized pathology reports and the electronic medical record. Etiologic classification was assigned based on the terminology in the pathology report, available special stains and immunohistochemical studies, molecular testing when performed, medication exposure, microbiologic evaluation, and documented clinical diagnoses. Cases were categorized as unknown when the available pathology report and clinical record did not support assignment to a specific reactive, infectious, medication-related, systemic, or neoplastic histiocytic disorder. Associated histiocytic disorders, such as LCH, Erdheim-Chester disease, and Rosai-Dorfman disease, were recorded, along with available molecular testing results for BRAF, MAP kinase pathway, and ALK alterations. Descriptive statistics were used to summarize demographic, clinical, endoscopic, and histopathologic findings. Continuous variables are reported as mean ± standard deviation, and categorical variables are presented as number and percentage.

**Table 1:** Practical diagnostic features of selected GI histiocytic entities AFB: acid-fast bacilli; CKD: chronic kidney disease; CNS: central nervous system; ESRD: end-stage renal disease; GI: gastrointestinal; PAS: periodic acid-Schiff

Entity/process	Typical GI pattern	Helpful stains/markers	Clinical clue
Langerhans cell histiocytosis	Polyps, ulcers, mucosal infiltrates	CD1a, langerin/CD207, S100; BRAF/MAP2K1 testing	Multifocal/systemic disease
Rosai-Dorfman disease	Rare GI involvement	S100+, CD68+, CD163+, emperipolesis; CD1a negative	Nodal/extranodal disease
Erdheim-Chester disease	Rare digestive involvement	CD68+, CD163+, factor XIIIa+; CD1a negative; BRAF possible	Bone, retroperitoneal, vascular/CNS disease
Histiocytic sarcoma	Mass-forming lesion	CD68, CD163, lysozyme; exclusion of carcinoma/lymphoma	Aggressive neoplasm
Xanthoma/xanthelasma	Foamy mucosal histiocytes	CD68+; PAS negative	Local inflammation, *Helicobacter pylori*, metabolic disease
Whipple disease	Lamina propria macrophages	PAS+, *Tropheryma whipplei *testing	Diarrhea, arthralgia, weight loss
Mycobacterial infection	Histiocytic/granulomatous inflammation	AFB/Fite positive	Immunosuppression, infection risk
Malakoplakia	Sheets of histiocytes	Michaelis-Gutmann bodies; von Kossa/PAS may help	Chronic infection/immunosuppression
Melanosis/pseudomelanosis	Pigmented macrophages	Iron/hemosiderin stains depending on context	Laxative use, iron, CKD, bleeding
Lanthanum deposition	Histiocytic deposits with foreign material	Histology ± elemental analysis	ESRD, lanthanum use

## Results

Cleveland Clinic clinical case series

The review of Cleveland Clinic patients for pathology of histiocytes in the GI tract yielded 108 cases. Sixty-five patients (60.2%) were identified as female, and the average age was 63.7 years. Seventy-one patients (65.7%) were White individuals, and 30 patients (27.8%) were Black individuals. Presenting symptoms included abdominal pain in 26 patients (24.1%), heartburn in 19 (17.6%), and dysphagia in 10 (9.3%). Overall, 67 patients (62.0%) had symptoms or signs prompting endoscopy, with anemia being the leading sign in 16 patients (14.8%). Thirty-eight patients (35.2%) were asymptomatic, and symptom status was not clearly documented in three patients (2.8%). Sixty-two cases (57.4%) involved the stomach, 33 (30.6%) involved the small intestine, and 14 (13.0%) involved the large intestine. Morphology on endoscopy included erosions in seven patients (6.5%), ulcers in seven (6.5%), nodules in 11 (10.2%), polyps in 19 (17.6%), and plaques in two (1.9%). Other findings seen on esophagogastroduodenoscopy (EGD) were mucosal changes ranging from erythema, friability, and atrophic changes to normal mucosa. The most common etiology of GI histiocytes for Cleveland Clinic patients was xanthomas or xanthelasma, occurring in 32 patients (29.6%). There were two cases (1.9%) of Whipple disease, one involving the duodenum and one involving the stomach. Hemosiderin-containing histiocytes suggestive of prior GI bleed were present in three cases (2.8%), all of which were in the small bowel. There were four pigmented aggregate cases, including two cases (1.9%) of pseudomelanosis duodeni and two cases of melanosis coli. In two cases, lanthanum, a non-calcium, non-aluminum phosphate binder, led to deposition of histiocytes in the GI tract. In 53 cases (49.1%), the etiology was unknown and could not be determined based on the information available. Most unclassified cases likely reflected incidental histologic detection without subsequent standardized immunohistochemical, molecular, microbiologic, or systemic evaluation, particularly when lesions were small, asymptomatic, or interpreted as potentially reactive (Table 2).

**Table 2:** Demographic, clinical, endoscopic, and histopathologic characteristics of 108 adult patients with gastrointestinal (GI) histiocytosis identified from the Cleveland Clinic pathology database between 2007 and 2022 * Some patients had more than one reason for endoscopy; ^#^ Some patients had multiple sites of involvement.

Patient demographics	Cleveland Clinic (n = 108)
Female, n (%)	65 (60.2)
Male, n (%)	43 (39.8)
White individuals, n (%)	71 (65.7)
Black individuals, n (%)	30 (27.8)
Asian individuals, n (%)	3 (2.8)
Multiracial, n (%)	1 (0.9)
Race not available, n (%)	3 (2.8)
Age, years (mean ± SD)	63.74 ± 15.61
Presenting symptoms	Cleveland Clinic (n = 108)
Asymptomatic, n (%)	38 (35.2)
Abdominal pain, n (%)	26 (24.1)
Heartburn, n (%)	19 (17.6)
Diarrhea, n (%)	4 (3.7)
Dysphagia, n (%)	10 (9.3)
Melena or hematochezia, n (%)	7 (6.5)
Constipation, n (%)	0
Other	4 (3.7)
Indication for endoscopy	Cleveland Clinic (n = 113*)
Signs and symptoms, n (%)	70 (61.9)
Anemia, n (%)	16 (14.2)
Routine screening, n (%)	3 (2.7)
Crohn’s disease, n (%)	5 (4.4)
Follow-up and surveillance, n (%)	10 (8.8)
Other, n (%)	9 (8.0)
Location of histiocytosis	Cleveland Clinic (n = 114^#^)
Esophagus, n (%)	1 (0.9)
Stomach, n (%)	62 (54.4)
Small intestine, n (%)	33 (28.9)
Large intestine, n (%)	14 (12.3)
Liver, n (%)	1 (0.9)
Bile duct, n (%)	1 (0.9)
Pancreatic duct, n (%)	1 (0.9)
Portal lymph node	1 (0.9)
Morphology	Cleveland Clinic (n = 108)
Erosions, n (%)	7 (6.5)
Ulcers, n (%)	7 (6.5)
Nodules, n (%)	11 (10.2)
Polyps, n (%)	19 (17.6)
Plaques, n (%)	2 (1.9)
Other, n (%)	62 (57.4)
Etiology	Cleveland Clinic (n = 108)
Xanthoma, xanthelasma, n (%)	32 (29.6)
Langerhans cell histiocytosis, n (%)	0
Unknown, n (%)	53 (49.1)
Whipple disease, n (%)	2 (1.9)
Histiocytic sarcoma, n (%)	0
Prior GI bleed, n (%)	3 (2.8)
Pseudomelanosis duodeni, n (%)	2 (1.9)
Melanosis coli, n (%)	2 (1.9)
Lanthanum induced, n (%)	2 (1.9)
Reactive changes, n (%)	0
Other, n (%)	12 (11.1)

Index case presentation

**Figure 1:**
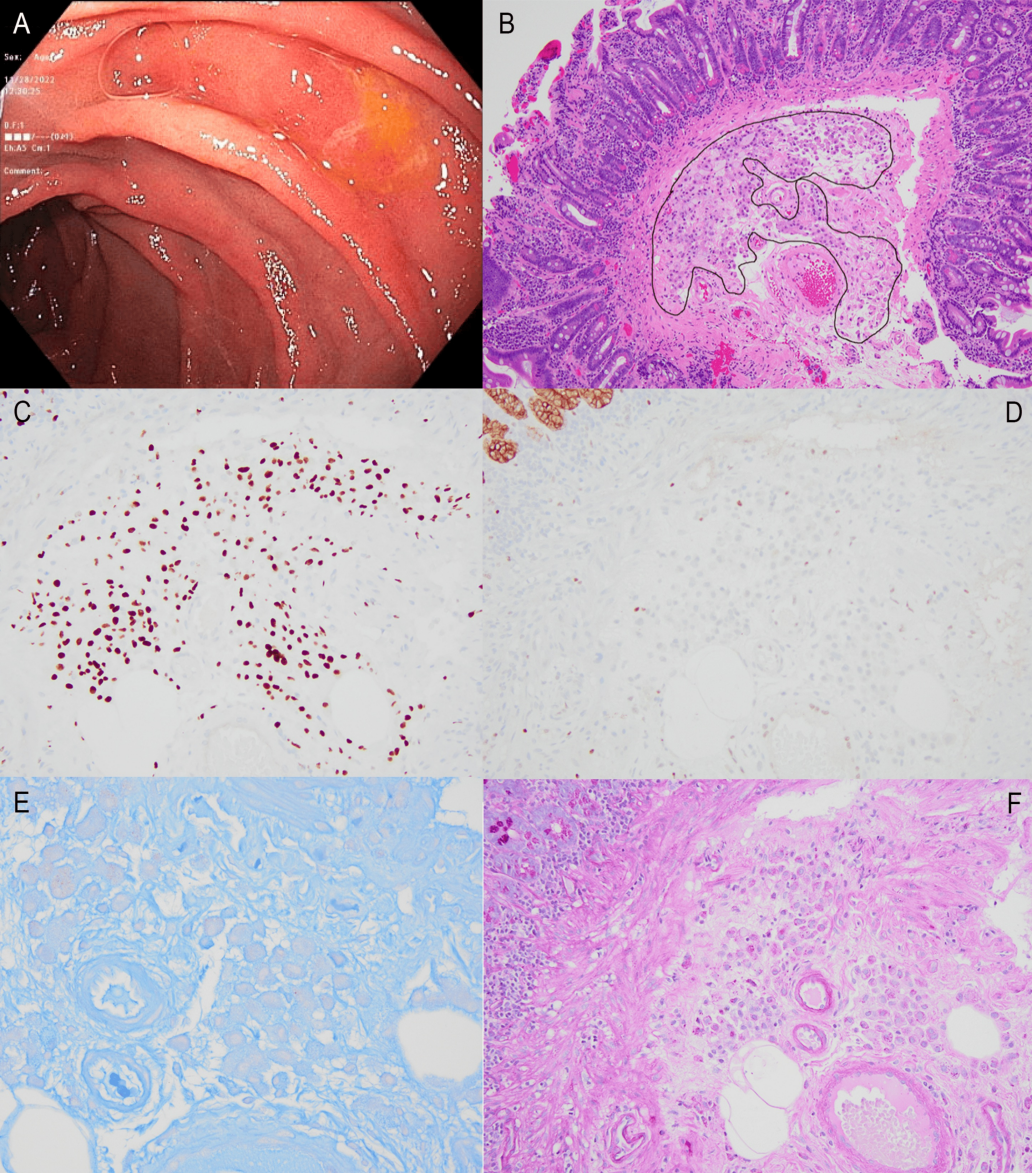
Composite endoscopic and histopathologic evaluation of incidental duodenal gastrointestinal histiocytosis identified in a 69-year-old male undergoing upper endoscopy and colonoscopy for gastroesophageal reflux symptoms (A) Upper endoscopy demonstrating a small sessile lesion in the second portion of the duodenum. (B) Hematoxylin and eosin-stained section of the duodenal biopsy showing a submucosal aggregate of histiocytes without evidence of epithelial dysplasia or malignancy. (C) Immunohistochemical staining demonstrating positive nuclear expression of PU.1 within the histiocytic infiltrate, supporting macrophage lineage. (D) Immunohistochemical staining negative for cytokeratin AE1/AE3, excluding epithelial differentiation. (E) Acid-fast bacteria (AFB)/Fite stain negative for acid-fast organisms, arguing against mycobacterial infection. (F) Periodic acid-Schiff (PAS) stain negative for intracytoplasmic microorganisms, helping exclude Whipple disease and other PAS-positive infectious etiologies (Original magnification: 40x for panels B-F).

A 69-year-old male presenting with symptoms of gastroesophageal reflux underwent diagnostic EGD and screening colonoscopy. The EGD revealed a single 5 mm sessile polyp in the second portion of the duodenum (Figure 1A), which was biopsied. Colonoscopy demonstrated two 5 mm sessile polyps, one in the transverse colon and the other in the ascending colon. Both polyps were removed with cold biopsy forceps.

Pathology of the duodenal lesion demonstrated duodenal mucosa with submucosal collection of histiocytes (Figure 1B), with no evidence of mucosal dysplasia. Immunohistochemical stains performed on the biopsy specimen confirmed the presence of histiocytes by positive nuclear expression of PU.1 (Figure 1C) and negative cytokeratin AE1/AE3 expression (Figure 1D). Acid-fast bacteria (AFB)/Fite (Figure 1E) and PAS (Figure 1F) special stains were both negative for the presence of microorganisms. Pathology of the ascending colon polyp demonstrated a sessile serrated polyp, with no evidence of dysplasia. Microscopic examination of the transverse colon polyp showed colonic mucosa with no significant abnormality or dysplasia.

The patient underwent genomic analysis specifically looking for targetable mutations in BRAF, MAP kinase pathway, and ALK. The patient was referred to hematology for further evaluation. The genomic panel did not identify targetable BRAF, MAP kinase pathway, or ALK alterations. Hematology evaluation did not reveal systemic histiocytic disease, and observation was recommended.

## Discussion

This study represents the largest single-center cohort of GI histiocytosis reported to date and provides real-world insight into the clinical and histopathologic spectrum of this uncommon finding. Our review of Cleveland Clinic pathology records identified 108 cases over a 15-year period, suggesting that GI histiocytosis may be more common than previously appreciated. These lesions are often identified incidentally on biopsy and may remain underrecognized, particularly in the absence of overt systemic manifestations or dedicated diagnostic evaluation.

Literature review

**Figure 2:**
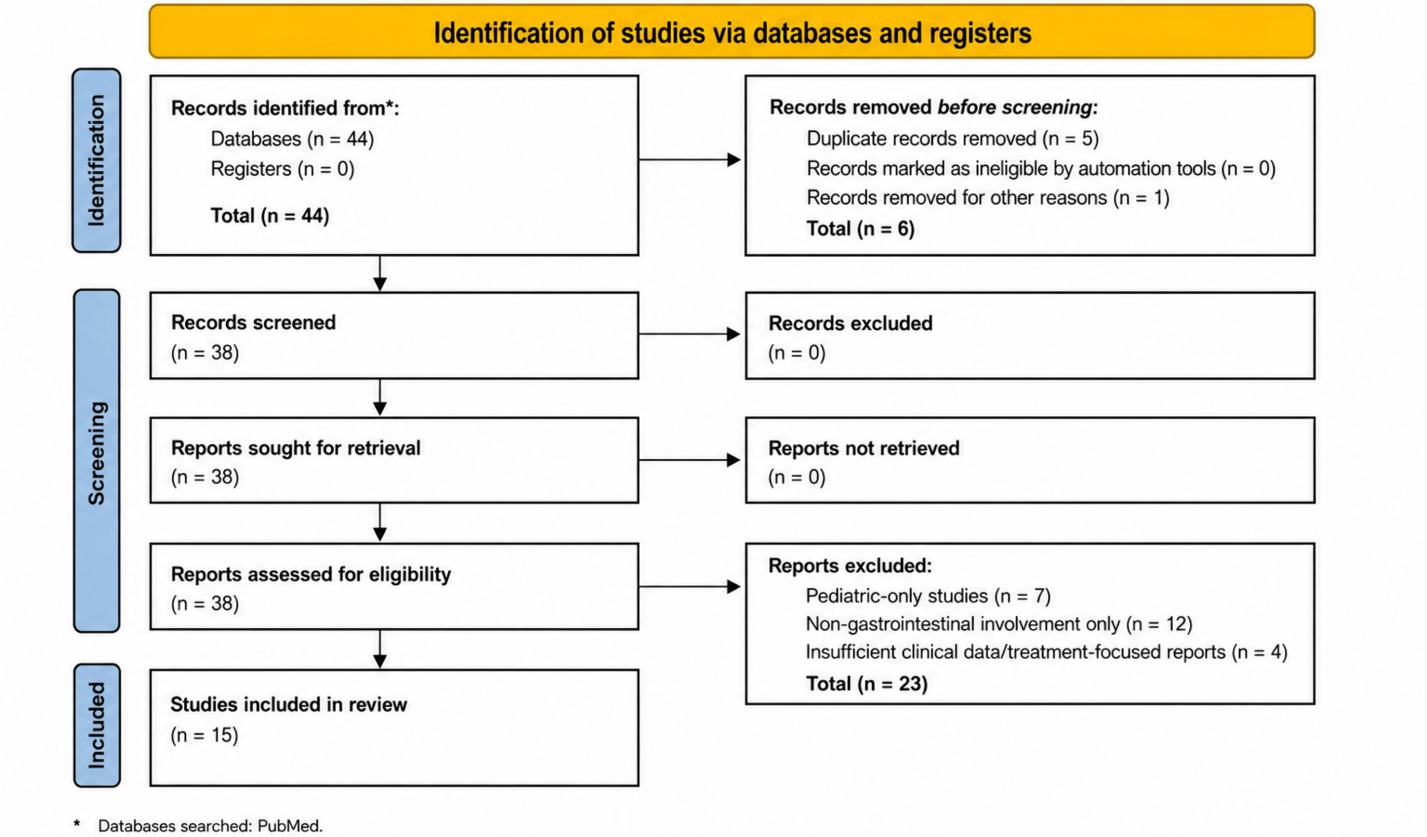
PRISMA flow diagram summarizing study selection for the literature review of published adult gastrointestinal histiocytosis cases A total of 44 records were identified through PubMed database searching. After screening and eligibility assessment, 15 studies met inclusion criteria and were included in the qualitative synthesis.

To contextualize our institutional findings, we performed a Preferred Reporting Items for Systematic Reviews and Meta-Analyses (PRISMA) 2020-guided review of published adult cases of GI histiocytosis reported between 2002 and 2022. Following screening and eligibility assessment, 15 studies comprising 26 adult cases were included in the qualitative synthesis. The PubMed search was performed on 1/15/2023 using the following search strategy: (“gastrointestinal” OR “esophagus” OR “stomach” OR “small intestine” OR “colon” OR “rectum” OR “liver” OR “gallbladder” OR “pancreas”) AND (“histiocyte” OR “histiocytosis” OR “Langerhans cell histiocytosis” OR “Rosai-Dorfman” OR “Erdheim-Chester” OR “histiocytic sarcoma” OR “xanthoma” OR “malakoplakia” OR “Whipple disease”). Studies were included if they involved adult patients (≥18 years) with histiocytosis affecting the GI tract, including the oropharynx to the anus, liver, gallbladder, or pancreas. Studies involving pediatric patients, disease limited to non-GI organs, duplicate reports, or articles focused exclusively on treatment outcomes or disease progression were excluded. Of the 44 articles initially identified, 29 were excluded after title, abstract, and full-text review based on predefined eligibility criteria, leaving 15 studies for final inclusion in the literature review. The study selection process is summarized in Figure 2.

A total of 26 published cases were identified, most of which represented LCH diagnosed after extensive immunohistochemical and molecular evaluation. Compared with our institutional cohort, published cases were more likely to represent clinically significant or extensively investigated disease, highlighting the potential publication bias toward rare neoplastic histiocytic disorders. The literature review yielded 26 cases of GI histiocytosis. Similar to CCF patients, there was a female predominance (69.23%) with an average age of 54.5 years. Racial data were not available for the majority (76.92%) of these patients. Again, similar to Cleveland Clinic patients, 10 (38.46%) patients were asymptomatic, followed by four patients (15.38%) with abdominal pain. The indication for endoscopy was based on signs and symptoms in 14 of the cases (53.85%). The most common site of involvement was one or more parts of the large intestine in 15 cases (51.72%). The remaining cases involved the esophagus, stomach, and small intestine. Endoscopy demonstrated polyps (42.31%), ulcers (26.92%), and nodules (7.69%). In contrast to Cleveland Clinic patients, no plaques, erosions, or general mucosal changes were reported. Four patients (15.38%) had positive BRAF mutations. Three patients (11.54%) were negative for the BRAF mutation. The status of BRAF mutations was not reported for the remaining 19 patients (73.08%). The most common etiology was LCH among 21 of the 26 cases (80.77%). Three cases (11.54%) were attributed to histiocytic sarcoma, a rare malignancy that accounts for <1% of hematologic neoplasms and usually presents with lymph node involvement, although the skin and GI tract can be involved [17]. In one patient (3.85%), lanthanum led to deposition of histiocytes in the esophagus, stomach, and small bowel [18]. A case report has described lanthanum-induced histiocytosis in the stomach and duodenum of a patient with end-stage renal disease, evidenced by biopsies with aggregates of basophilic material and heavy metals on transmission electron microscopy [18].

Across both cohorts, patients were predominantly middle-aged and female, and a substantial proportion were asymptomatic at presentation. Although female patients comprised 60.2% of the institutional cohort, no clear sex-specific pattern in presentation, anatomic distribution, or etiologic category was identified on descriptive review. When symptoms were present, they were nonspecific and overlapped with common GI complaints such as abdominal pain, heartburn, dysphagia, and anemia. Endoscopic findings were similarly heterogeneous, ranging from normal-appearing mucosa to polyps, nodules, ulcers, and plaques, underscoring that no single endoscopic pattern reliably predicts histiocytic pathology. These findings highlight why GI histiocytosis can be easily overlooked or dismissed as an incidental histologic finding without further evaluation.

A major distinction between the Cleveland Clinic cohort and previously published cases is the degree of etiologic attribution. In the literature, most reported cases were ultimately classified as LCH, typically after extensive immunohistochemical and molecular evaluation. In contrast, nearly half of cases in our institutional series remained of unknown etiology. This difference likely reflects variation in diagnostic intensity rather than underlying disease biology, as many institutional cases were incidental findings that did not trigger further investigation. These findings suggest that GI histiocytosis often resides in a diagnostic gray zone-identified histologically but not systematically evaluated clinically, particularly in the absence of systemic features. Xanthomatous histiocytic findings may represent benign reactive changes related to local inflammation, metabolic disease, medication exposure, or prior mucosal injury. However, in selected cases, particularly when lesions are multifocal, mass-forming, ulcerated, associated with symptoms, or lack a clear reactive explanation, these findings may raise concern for a broader histiocytic disorder requiring further evaluation. Therefore, additional workup should be guided by the clinical context, lesion morphology, distribution, systemic features, and available histopathologic findings.

Prognosis

The outcomes for patients with GI histiocytosis vary depending on the etiology. LCH has a better prognosis in adults than in children, as the pediatric population often has systemic disease [1]. GI involvement in LCH usually occurs in children with systemic disease and therefore is associated with worse outcomes, while adult GI LCH is extremely rare with limited data on outcomes [19]. In Erdheim-Chester disease, involvement of digestive organs (e.g., pancreas, liver, GI tract, gallbladder) is associated with worse outcomes (hazard ratio, 4.74; 95% CI, 1.05-21.4; P=0.043) [20]. Rosai-Dorfman is a benign condition that usually spontaneously resolves; however, cases with GI involvement have a protracted course without spontaneous resolution, and liver involvement is specifically associated with a worse clinical outcome [21,22]. Among the primary histiocytic disorders of neoplastic origin, histiocytic sarcoma has a poor prognosis with a median survival of six months, while follicular dendritic sarcoma is a more indolent malignancy with two-year and five-year survival at 82% and 79%, respectively [23,24]. Xanthomas and xanthelasmas are benign lesions associated with inflammation, *Helicobacter pylori* infection, diabetes, and hyperlipidemia [25]. However, studies have shown that gastric xanthelasma is a predictive marker for gastric cancer development [25]. As Whipple disease is an infection, most patients have symptomatic improvement in one to three weeks with proper antibiotic treatment, although 17-35% of patients have been reported to relapse [26]. Patients with Whipple disease who require hospitalization most commonly do so because of GI manifestations of the disease [27]. The prognosis of Tangier disease depends on the progression of atherosclerosis [28]. As there are several lysosomal storage diseases, the prognosis varies depending on disease phenotype, early diagnosis, and specific treatment including enzyme replacement therapy, anti-inflammatory agents, stem cell transplant, and gene therapy [29]. Melanosis coli typically resolves within one year of laxative cessation [15]. The prognostic significance of pseudomelanosis duodeni is unknown; however, resolution after discontinuation of oral iron occurred in patients with β-thalassemia, chronic kidney disease, diabetes, and hypertension [30].

Outcomes

Among the 108 CCF cases, patients were followed for an average of 583.5 days with a standard deviation of 58.69 days from the date of endoscopy that identified histiocytosis to follow up with a gastroenterologist or death. Fifty-eight (53.70%) of the 108 patients had follow-up with GI, although only six (5.56%) were specifically further evaluated for the GI histiocytosis. This limited downstream evaluation likely reduced the ability to determine definitive etiology and may have underestimated clinically relevant associated conditions. Among the six that were further evaluated, one patient had an inconclusive diagnosis, three were referred to hematology/oncology, one was referred to infectious disease for colonic histoplasmosis, and one was diagnosed with lanthanum-induced histiocytosis. Eight (7.41%) patients were lost to follow-up, and 17 (15.74%) were deceased when tracking outcomes. The case series of Cleveland Clinic patients demonstrates that GI histiocytosis may be more common than previously thought. Based on the data from Cleveland Clinic patients, it is also possible that the incidental histiocyte lesions are not undergoing complete diagnostic workup.

Limitations

This study has several limitations. First, its retrospective design and reliance on pathology database queries introduce potential selection and referral bias, particularly given the quaternary-care setting. Second, etiologic classification was limited by incomplete clinical, laboratory, and molecular evaluation in many cases, which likely contributed to the high proportion of histiocytosis deemed idiopathic. Third, molecular testing for BRAF, MAP kinase pathway alterations, and ALK rearrangements was not performed uniformly and was often guided by clinical suspicion rather than standardized criteria. Fourth, race and ethnicity data were missing for a substantial proportion of patients in the literature review, limiting comparative demographic analyses. Fifth, the predominance of LCH in published reports likely reflects publication bias, because unusual clonal or neoplastic histiocytic disorders are more likely to be reported than incidental reactive histiocytic aggregates encountered in routine biopsy practice. Additionally, centralized rereview of all histopathology slides was not performed; therefore, classification relied on finalized pathology reports and available ancillary testing. Finally, true disease incidence cannot be inferred from this pathology-based cohort, and outcomes may not be generalizable to community practice. Despite these limitations, the large institutional cohort provides important real-world insight into the spectrum, evaluation gaps, and clinical relevance of GI histiocytosis.

Approach to incidental GI histiocytosis

A practical approach to incidental GI histiocytosis should begin with correlation between the endoscopic appearance, histologic pattern, and clinical context. For small incidental xanthomatous or pigmented aggregates without concerning symptoms, a focused review of medication exposures, prior bleeding, laxative use, renal disease, lipid disorders, diabetes, and local inflammatory conditions may be sufficient. When histiocytic infiltrates are mass-forming, multifocal, ulcerated, associated with systemic symptoms, or lack a clear reactive explanation, additional evaluation should include targeted special stains for infectious etiologies, immunohistochemistry to define histiocytic lineage and exclude epithelial or lymphoid mimics, and selective molecular testing for alterations such as BRAF, MAP kinase pathway mutations, or ALK rearrangements. Referral to hematology/oncology should be considered for patients with systemic manifestations, multifocal disease, atypical histology, or molecular findings suggestive of a clonal histiocytic disorder.

## Conclusions

In summary, GI histiocytosis encompasses a broad spectrum of etiologies, most of which are individually rare. Patients may present with nonspecific GI symptoms, including abdominal pain, heartburn, dysphagia, and diarrhea, although more than one-third are asymptomatic at diagnosis. Involvement can occur throughout the GI tract, from the esophagus to the rectum, with endoscopic findings ranging from subtle mucosal changes to polyps, nodules, and ulcerations. Our findings suggest that GI histiocytosis may be more common than previously recognized but frequently remains underreported due to incomplete diagnostic evaluation. Incidental histiocytic aggregates should not automatically be dismissed as clinically irrelevant; rather, they should prompt a context-dependent assessment for local inflammation, infection, medication-related deposition, prior hemorrhage, metabolic disease, or, less commonly, an underlying clonal histiocytic disorder. Careful clinical correlation, combined with detailed endoscopic and histopathologic assessment and selective molecular testing, is essential to accurately identify underlying etiologies and distinguish incidental findings from clinically significant histiocytic disorders.

## References

[1] Singhi AD, Montgomery EA (2011). Gastrointestinal tract langerhans cell histiocytosis: a clinicopathologic study of 12 patients. Am J Surg Pathol.

[2] Cline MJ (1994). Histiocytes and histiocytosis. Blood.

[3] Bhinder J, Mori A, Kurtz L, Reddy M (2018). Langerhans cell histiocytosis of the gastrointestinal tract - a rare entity. Cureus.

[4] Detlefsen S, Fagerberg CR, Ousager LB, Lindebjerg J, Marcussen N, Nathan T, Sørensen FB (2013). Histiocytic disorders of the gastrointestinal tract. Hum Pathol.

[5] Wang L, Yang F, Ding Y (2022). Gastrointestinal Langerhans cell histiocytosis with unifocal, single-system involvement in adults: cases report and literature review. J Clin Lab Anal.

[6] Alayed K, Medeiros LJ, Patel KP (2016). BRAF and MAP2K1 mutations in Langerhans cell histiocytosis: a study of 50 cases. Hum Pathol.

[7] Kemps PG, Picarsic J, Durham BH (2022). ALK-positive histiocytosis: a new clinicopathologic spectrum highlighting neurologic involvement and responses to ALK inhibition. Blood.

[8] Chatterjee A, de la Fuente J, Rech KL, Takahashi N, Majumder S (2023). A rare cause of abdominal pain: Erdheim-Chester disease. ACG Case Rep J.

[9] Arnaud L, Gorochov G, Charlotte F (2011). Systemic perturbation of cytokine and chemokine networks in Erdheim-Chester disease: a single-center series of 37 patients. Blood.

[10] Dehner LP (2003). Juvenile xanthogranulomas in the first two decades of life: a clinicopathologic study of 174 cases with cutaneous and extracutaneous manifestations. Am J Surg Pathol.

[11] Afonso JP, Ando PN, Padilha MH, Michalany NS, Porro AM (2013). Cutaneous malakoplakia: case report and review. An Bras Dermatol.

[12] Kolovou GD, Mikhailidis DP, Anagnostopoulou KK, Daskalopoulou SS, Cokkinos DV (2006). Tangier disease four decades of research: a reflection of the importance of HDL. Curr Med Chem.

[13] Röyttä M, Fagerlund AS, Toikkanen S, Salmi TT, Jorde LB, Forsius HR, Eriksson AW (1992). Wolman disease: morphological, clinical and genetic studies on the first Scandinavian cases. Clin Genet.

[14] Benavides SH, Morgante PE, Monserrat AJ, Zarate J, Porta EA (1997). The pigment of melanosis coil: a lectin histochemical study. Gastrointest Endosc.

[15] Tian C, Al Nasser Y (2025). Melanosis coli. StatPearls [Internet].

[16] Jung IS, Kim JO, Lee JS, Lee MS, Shim CS (2003). Barium granuloma of rectum diagnosed by EUS. Gastrointest Endosc.

[17] Lio J, Velazquez AI, Angelino K, Kasmin F (2017). Multifocal gastric histiocytic sarcoma presenting as gastrointestinal bleeding. Am J Gastroenterol.

[18] Rothenberg ME, Araya H, Longacre TA, Pasricha PJ (2015). Lanthanum-induced gastrointestinal histiocytosis. ACG Case Rep J.

[19] Matsuoka Y, Iemura Y, Fujimoto M (2021). Upper gastrointestinal Langerhans cell histiocytosis: a report of 2 adult cases and a literature review. Int J Surg Pathol.

[20] Toya T, Ogura M, Toyama K (2018). Prognostic factors of Erdheim-Chester disease: a nationwide survey in Japan. Haematologica.

[21] Alatassi H, Ray MB, Galandiuk S, Sahoo S (2006). Rosai-Dorfman disease of the gastrointestinal tract: report of a case and review of the literature. Int J Surg Pathol.

[22] Anders RA, Keith JN, Hart J (2003). Rosai-Dorfman disease presenting in the gastrointestinal tract. Arch Pathol Lab Med.

[23] Li L, Shi YH, Guo ZJ (2010). Clinicopathological features and prognosis assessment of extranodal follicular dendritic cell sarcoma. World J Gastroenterol.

[24] Kommalapati A, Tella SH, Durkin M, Go RS, Goyal G (2018). Histiocytic sarcoma: a population-based analysis of incidence, demographic disparities, and long-term outcomes. Blood.

[25] Moumin FA, Mohamed AA, Osman AA, Cai J (2020). Gastric xanthoma associated with gastric cancer development: an updated review. Can J Gastroenterol Hepatol.

[26] Durand DV, Lecomte C, Cathébras P, Rousset H, Godeau P (1997). Whipple disease. Clinical review of 52 cases. The SNFMI Research Group on Whipple Disease. Société Nationale Française de Médecine Interne. Medicine (Baltimore).

[27] Ahmad AI, Wikholm C, Pothoulakis I, Caplan C, Lee A, Buchanan F, Kyoo Cho W (2022). Whipple's disease review, prevalence, mortality, and characteristics in the United States: a cross-sectional national inpatient study. Medicine (Baltimore).

[28] Alshaikhli A, Vaqar S (2023). Tangier disease. StatPearls [Internet].

[29] Rajkumar V, Dumpa V (2023). Lysosomal storage disease. StatPearls [Internet].

[30] Felipe-Silva A, de Campos FP, da Silva JG (2011). Duodenal pseudomelanosis (pseudomelanosis duodeni): a rare endoscopic finding. Autops Case Rep.

